# Assessing Lemon Peel Waste as a Solid Biofuel: A Study of Its Combustion Behaviour, Kinetics, and Thermodynamics

**DOI:** 10.3390/polym17212830

**Published:** 2025-10-23

**Authors:** Mohamed Anwar Ismail, Ibrahim Dubdub, Suleiman Mousa, Mohammed Al-Yaari, Majdi Ameen Alfaiad, Abdullah Alshehab

**Affiliations:** 1 Mechanical Engineering Department, King Faisal University, P.O. Box 380, Al-Ahsa 31982, Saudi Arabia; maismail@kfu.edu.sa; 2 Chemical Engineering Department, King Faisal University, P.O. Box 380, Al-Ahsa 31982, Saudi Arabiamalyaari@kfu.edu.sa (M.A.-Y.);; 3 Physics Department, King Faisal University, P.O. Box 380, Al-Ahsa 31982, Saudi Arabia

**Keywords:** lemon peel, combustion, solid biofuel, thermogravimetric analysis (TGA), kinetics analysis, model-free, thermodynamics

## Abstract

This study provides a comprehensive analysis of lemon peel (LP) combustion behaviour using combined physicochemical characterization and non-isothermal thermogravimetric kinetics. To achieve this, LP was characterized for its proximate and ultimate composition, with its structure analysed via FTIR, XRD, and SEM. Thermogravimetric analysis (TGA) was then performed at high heating rates (20–80 K min^−1^) to investigate combustion stages, and kinetic and thermodynamic parameters were determined using six model-free and one model-fitting method. The results revealed a high heating value (23.02 MJ kg^−1^) and high volatile matter (73.2 wt%), establishing LP’s significant energy potential. TGA displayed four distinct decomposition stages corresponding to dehydration, pectin/hemicellulose, cellulose, and lignin/char combustion. Kinetic analysis yielded activation energies that varied with conversion, peaking at approximately 304 kJ mol^−1^, and a three-dimensional diffusion (D3) mechanism was identified as the rate-limiting step. In conclusion, while its high energy content and low nitrogen (1.26 wt%) and sulphur (0.20 wt%) content make LP an attractive low-emission biofuel, its viability is challenged by a high potassium concentration in the ash (34.8 wt% K_2_O), posing a severe risk of slagging. This study provides the comprehensive combustion kinetic data for LP at high heating rates, which is essential for designing appropriate energy conversion technologies and ash management strategies.

## 1. Introduction

The global pursuit of sustainable energy is intensifying as nations seek to mitigate climate change and reduce dependence on finite fossil fuels [1]. In this transition, biomass has emerged as a key renewable and carbon-neutral resource [2], with recent reviews highlighting the significant potential of harnessing fruit and vegetable waste for biofuel production [3]. Agro-industrial wastes, in particular, represent a valuable and underutilized feedstock. Valorizing these materials not only provides a source of clean energy but also addresses the environmental challenges of waste management, contributing to a more circular economy [4], a concept that is central to the modern valorization of citrus waste into products ranging from biofuels to advanced materials [5,6]. Indeed, citrus peels are now widely explored as a sustainable bioresource for producing a diverse range of products, from engine fuel additives and industrial chemicals to advanced materials like biodegradable packaging films [7,8,9]. As lemon peel is a complex lignocellulosic matrix, rich in the biopolymers pectin, cellulose, and hemicellulose, a detailed study of its thermal stability and kinetic behavior is of interest to the field of cellulose and wood-based composite technologies.

Among these resources, citrus waste is generated in vast quantities worldwide, with annual production estimated to be close to 10 million metric tons [10]. The lemon processing industry, for example, discards 40–60% of the fruit’s mass as peel, pulp, and seeds [11,12]. Due to its composition, LP is a promising candidate for biofuel production [13,14], and its significant energy content, highlighted by a high heating value, makes it suitable for thermochemical conversion processes such as combustion and gasification [15].

Previous investigations into the thermochemical behavior of LP have explored several valorization pathways. A significant portion of research has focused on pyrolysis under inert atmospheres. For instance, multiple studies have explored the pyrolytic decomposition of LP and other citrus wastes to produce bio-oil and other products [16,17]. A common approach involves converting the raw peel into biochar via slow pyrolysis, which can then be used as a solid fuel or a functional material like magnetic biochar for wastewater treatment [18,19]. Other uses, such as the direct agronomic application of citrus waste as a soil amendment, have also been investigated [20]. While these pyrolysis studies and their kinetic analyses [21] are valuable, and other pathways like bio-ethanol and biogas production are also being explored [6], a significant knowledge gap remains concerning the direct combustion of raw LP. More specifically, its behavior under the high heating rates (>20 K min^−1^) characteristic of industrial boilers and thermal reactors has not been explored. Understanding these direct combustion dynamics is essential for designing and optimizing practical energy recovery systems for this abundant waste stream.

This study aims to fill this critical gap by providing the first comprehensive analysis of LP combustion at industrially relevant heating rates. Building on previous work on mango peel [22] and orange peel [23], the primary objectives are: (1) to perform a thorough physicochemical, structural, and morphological characterization of LP; (2) to investigate its combustion behavior at high heating rates (20, 40, 60, and 80 K min^−1^) using thermogravimetric analysis (TGA); (3) to conduct a rigorous kinetic analysis using six model-free methods and the Coats–Redfern model-fitting method; and (4) to calculate key thermodynamic parameters to assess the reaction’s feasibility. This work provides an essential dataset for evaluating the potential of LP as a sustainable solid biofuel and for the design of efficient combustion technologies.

## 2. Materials and Methods

### 2.1. Lemon Peel Collection and Sample Preparation

The preparation protocol, which involved washing, drying at 310 K for 24 h, grinding, and sieving to a uniform particle size of 0.34 ± 0.05 mm, was consistent with methods established in prior research on other citrus and mango wastes [22,23]. Fresh peels from lemons (*Citrus limon*) were procured from a local market in Al-Ahsa, Saudi Arabia. Upon collection, the material was first rinsed with distilled water to eliminate surface contaminants and then manually separated. To reduce the initial moisture content, the raw peels were subjected to drying for 24 h in a hot air oven (Memmert UN110, Büchenbach, Germany) set to 310 K. The dried material was subsequently pulverized using a laboratory mill (IKA MF 10 Basic, Staufen, Germany) and passed through an ELE Sieve Shaker (ELE International, Milton Keynes, UK). This step was performed to obtain a uniform sample with a consistent particle size of 0.34 ± 0.05 mm. To protect against atmospheric moisture prior to analysis, the final LP powder was kept in sealed, airtight containers at 298 K.

### 2.2. Physico-Chemical Characterization

#### 2.2.1. Proximate Analysis

The proximate composition of the lemon peel (LP), including its moisture, volatile matter, and ash content, was determined using a Mettler Toledo TGA-2 Star System (TGA-2 Star System, Mettler Toledo, Greifensee, Switzerland) All measurements were performed in triplicate. For each analysis, a sample mass of approximately 10 mg was subjected to a multi-stage heating program under a controlled atmosphere. Moisture content was measured by heating the sample to 383 K for 30 min in a nitrogen environment. Subsequently, the volatile matter was quantified by increasing the temperature to 1173 K for 7 min, also under nitrogen. Finally, the atmosphere was switched to oxygen, and the sample was held at 823 K for 15 min to determine the ash content. All measurements were performed in triplicate to ensure accuracy and reproducibility (SD < 0.5%). While ISO standards (e.g., ISO 18122 for ash, ISO 18123 for volatiles, ISO 18134 for moisture) represent the benchmark for commercial solid biofuel analysis, providing direct gravimetric principles, the TGA-based method used herein aligns conceptually with these standards and their ASTM equivalents (e.g., D3174 for ash). It offers comparable validity for research-scale characterization but is particularly advantageous for kinetic studies requiring small, homogeneous samples (~10 mg) and data under precisely controlled heating environments, ensuring consistency between proximate and combustion kinetic experiments [24,25]. The fixed carbon content was then calculated by difference, as shown in Equation (1):

(1) Fixed Carbon (%)=100−(Moisture + Volatile Matter + Ash) 

#### 2.2.2. Ultimate Analysis

The elemental composition (carbon, hydrogen, nitrogen, and sulfur) of the LP was determined simultaneously via ultimate analysis performed on a Vario EL III CHNS elemental analyzer (Elementar, Langenselbold, Germany). The analysis was conducted in triplicate. A small sample of approximately 2 mg was combusted at 1273 K in an oxygen-rich atmosphere. The resulting combustion gases were quantified using a thermal conductivity detector. The oxygen content was then calculated by difference on a dry basis, accounting for the ash content, as per Equation (2):

(2) Oxygen (%)=100−(C+H+N+S+ Ash)

#### 2.2.3. Fiber Analysis

To quantify the primary structural components, the Van Soest method was employed using an ANKOM 2000 Automated Fiber Analyzer (ANKOM Technology, Macedon, NY, USA). A 0.5 g sample of LP was subjected to sequential digestion. The sample was first treated with a neutral detergent solution to yield the neutral detergent fiber (NDF). This was followed by digestion in an acid detergent solution to determine the acid detergent fiber (ADF). Finally, 72% sulfuric acid was used to isolate the acid detergent lignin (ADL). From these fractions, the hemicellulose and cellulose contents were calculated using Equation (3):

(3)             Hemicellulose = NDF − ADFCellulose = ADF−ADL

#### 2.2.4. Higher Heating Value (HHV)

The energetic content of the biomass was quantified by measuring its Higher Heating Value (HHV) with a Plain Oxygen Bomb Calorimeter (Model 1341EE, Parr Instrument Company, Moline, IL, USA). A 0.5 g sample of LP was pelletized and then combusted in the calorimeter under an oxygen pressure of 3 MPa. The instrument was calibrated against a benzoic acid standard, and all measurements were performed in triplicate to ensure precision.

### 2.3. Structural, Elemental, and Morphological Characterization

#### 2.3.1. Fourier Transform Infrared (FTIR) Spectroscopy

The functional groups present in the LP biomass were identified via Fourier Transform Infrared (FTIR) spectroscopy using a JASCO FTIR-4000 spectrometer (Tokyo, Japan). For the analysis, a pellet was prepared by mixing approximately 2 mg of LP powder with 200 mg of potassium bromide (KBr) and pressing the mixture. The sample was scanned 32 times over a wavenumber range of 4000–400 cm^−1^ with a spectral resolution of 4 cm^−1^.

#### 2.3.2. X-Ray Diffraction (XRD) Analysis

The crystalline structure and mineral phases of the LP were investigated by X-ray Diffraction (XRD) on a Rigaku Ultima IV diffractometer (Akishima, Japan). The instrument was operated at 40 kV and 30 mA, using Cu Kα radiation (λ = 1.54 Å). For the analysis, the dried LP powder was tightly packed into a standard sample holder to ensure a flat, smooth surface. Samples were scanned across a 2θ range from 10° to 90° at a scan rate of 1° per minute and a step size of 0.02°. The crystallinity index (CrI) was determined from the diffraction pattern using the Segal method (Equation (4)):

(4) $$CrI(\%)=\left(\frac{I_{002}-I_{am}}{I_{002}}\right)\times 100$$

where $I_{002}$ is the intensity of the cellulose-I peak at $2\theta \approx 22^{\circ }$, and $I_{\mathrm{am}\;}$ is the intensity of the amorphous background at 2θ $\approx 18^{\circ }$.

#### 2.3.3. X-Ray Fluorescence (XRF) Analysis

The elemental composition of the LP ash was determined by X-ray Fluorescence (XRF) spectroscopy with a Lab-X3500 spectrometer (Oxford Instruments, Abingdon, UK). The ash was prepared in accordance with ASTM D3174-11 by combusting 5 g of the raw sample at 973 K. The resulting ash was then pressed into a pellet using a boric acid binder for analysis under vacuum. Macro elements (e.g., K, Ca, Si, S) were quantified using a standard less calibration method, with measurements conducted in triplicate.

#### 2.3.4. Scanning Electron Microscopy (SEM)

The surface morphology of the lemon peel powder was examined using a JEOL JSM-7600F Field Emission Scanning Electron Microscope (JEOL Ltd., Akishima, Japan). Samples were first mounted onto aluminum stubs using double-sided carbon tape. To ensure conductivity and prevent charging during imaging, the mounted samples were then sputter-coated with a thin layer of gold. The analysis was performed using a secondary electron detector (SEI) with an accelerating voltage of 15.0 kV and a working distance (WD) of approximately 8.1 mm. Micrographs were captured at various magnifications to observe both the general particle structure and fine surface details.

### 2.4. Thermogravimetric Analysis (TGA)

The combustion characteristics of LP were examined using a NETZSCH STA 449 F3 Jupiter simultaneous TG–DSC analyzer (NETZSCH-Gerätebau, Selb, Germany). In each experiment, a sample of approximately 10 mg was placed in an aluminum oxide (Al_2_O_3_) crucible. The sample was then heated from 303 K to 973 K under a synthetic air atmosphere (80% N_2_, 20% O_2_) with a constant flow rate of 60 mL/min. The experiments were conducted at four different linear heating rates: 20, 40, 60, and 80 K/min^−1^. Thermogravimetric (TG) and derivative thermogravimetric (DTG) curves were recorded in triplicate for data accuracy.

### 2.5. Kinetic and Thermodynamic Modelling

#### 2.5.1. Kinetic Analysis

To model the combustion kinetics, the experimental TGA data was processed using an established protocol where mass loss data is transformed into conversion data (α) for analysis over a specified range (e.g., α = 0.10 to 0.90), which is consistent with the prior studies [23,26]. The mass loss data from the four heating rates was transformed into conversion data (α), which was analyzed over the range of α = 0.10 to 0.90. The apparent activation energy, (*E_a_*), as a function of conversion was determined using six distinct isoconversional (model-free) methods: Friedman (FR), Flynn–Wall–Ozawa (FWO), Kissinger–Akahira–Sunose (KAS), Starink (STK), Kissinger (K), and the advanced Vyazovkin (VY) method, which was implemented using the software framework described by Drozin et al. (2020) [27]. Further details on these methods are provided in the Supplementary Materials (Table S1). Additionally, the Coats–Redfern (CR) model-fitting method was used to evaluate sixteen different solid-state reaction models to identify the most probable reaction mechanism (Table S2, Supplementary Materials).

#### 2.5.2. Thermodynamic Parameters

Key thermodynamic parameters for the activated complex formation during combustion—including activation enthalpy (Δ*H*), Gibbs free energy (Δ*G*), and entropy (Δ*S*)—were derived from the kinetic analysis. These values were calculated using the standard thermodynamic equations derived from transition state theory, presented below, which is consistent with the methodology used in previous work [22,23], presented below:

(5) $$∆H=E_{a}-R\;T_{p}\;$$

(6) $$∆G=E_{a}+R\;T_{p}ln\left(\frac{k_{B}\;T_{p}}{h\;A_{0}}\right)$$

(7) $$∆S=\frac{∆H-∆G}{T_{p}}$$

In these equations, *T_p_* is the peak temperature (K) from the DTG curve, *E_a_* is the activation energy, *R* is the universal gas constant, *k_B_* is the Boltzmann constant (1.381 × 10^−23^ J·K^−1^), *h* is the Planck constant (6.626 × 10^−34^ J·s), and *A*_0_ is the pre-exponential factor (min^−1^). The calculation of these parameters across the conversion range provides insight into the feasibility and energetic requirements of the LP combustion process.

## 3. Results and Discussion

### 3.1. Fundamental Properties of Lemon Peel

The physicochemical properties of the lemon peel (LP) sample are summarized in Table 1. The proximate analysis revealed a high volatile matter content (73.20 wt%), a desirable characteristic for biofuels as it promotes rapid ignition and efficient combustion. The ash content was moderate at 5.30 wt%, a value in excellent agreement with the 5.40 wt% reported by Pathak et al. (2017) [14]. This value is comparable to the 5.5 wt% that was previously reported for orange peel [23] but is notably lower than the 7.55 wt% found in mango peel [22], suggesting that ash management strategies will be a necessary, though perhaps less critical, consideration for LP compared to other fruit wastes. The volatile matter content of the LP is comparable to that of many agricultural residues like wheat straw (~70%) but slightly lower than that of woody biomass (~75–80%) [28,29]. The ash content (5.30 wt%) is significantly higher than that found in premium wood pellets (<1%) but is notably lower than high-ash agro-industrial wastes like rice husk (15–20%). From an energy perspective, the HHV of 23.02 MJ kg^−1^ is high, surpassing some of the agricultural residues like straw (~17.5 MJ kg^−1^) and many standard woody biomasses (~19–20 MJ kg^−1^) [28].

The ultimate analysis showed a high carbon content of 54.09 wt%, which contributes to its impressive Higher Heating Value (HHV) of 23.02 MJ kg^−1^. This energy content is superior to many agricultural residues and is significantly higher than the 21.9 MJ kg^−1^ that was measured for mango peel [22], positioning LP as a particularly energy-dense biofuel. The carbon content, and consequently the calculated oxygen (34.20 wt%), falls within the wide spectrum of values reported for lemon peel, which can range from 40.3 wt% to 46.1 wt% for carbon and 41.5 wt% to 52.3 wt% for oxygen. Such variations are common for biomass and are likely due to differences in lemon cultivar, geographical origin, and sample preparation methods across studies. Furthermore, the low nitrogen (1.26 wt%) and sulfur (0.20 wt%) contents are consistent with the literature [19] and are highly advantageous, indicating a significantly lower potential for the formation of harmful NO_x_ and SO_x_ emissions. LP’s elemental profile also compares favorably to common biofuels: its low N and S levels are a distinct advantage over high-N agro-wastes, while its carbon content (54.09 wt%) aligns with that of wood (45–55 wt%) [28,30]. This reinforces LP’s potential as a low-emission solid biofuel, although its high oxygen content (34.20 wt%) may influence combustion efficiency relative to denser woods.

The standard fiber analysis yielded very low values for hemicellulose (0.73%) and lignin (0.78%). This is a known limitation of the Van Soest method when applied to pectin-rich materials like citrus peels. Pectin, a major structural polysaccharide in LP, is largely solubilized during the analysis and thus not fully accounted for in the fiber fractions. As the thermal degradation of pectin occurs in a temperature range that overlaps with hemicellulose and cellulose, it is a critical component that will significantly influence the overall combustion profile.

### 3.2. Structural, Elemental, and Morphological Insights

#### 3.2.1. FTIR Spectroscopy: Functional Group Identification

**Figure 1:**
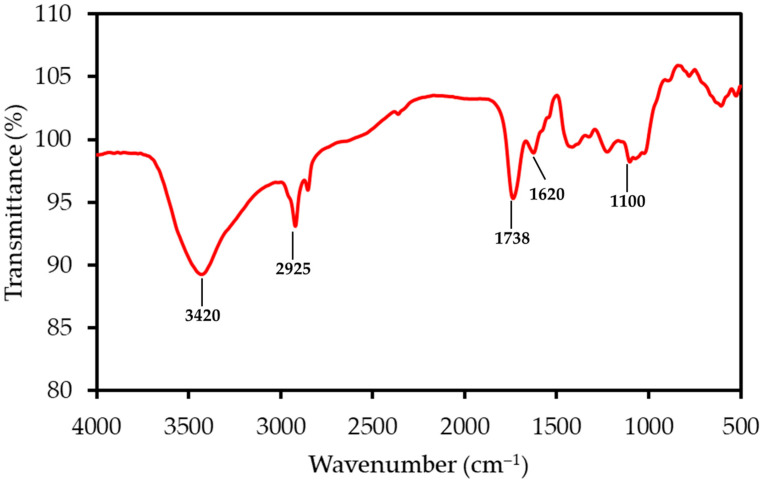
FTIR spectrum of the LP sample, with principal absorption bands annotated. The spectrum shows characteristic peaks for lignocellulosic materials, including hydroxyl (O-H), aliphatic (C-H), and carbonyl (C=O) functional groups.

The FTIR spectrum of LP, shown in Figure 1, reveals functional groups characteristic of its complex, polysaccharide-rich structure. A prominent and broad absorption band centered around 3350 cm^−1^ corresponds to the O-H stretching vibrations of hydroxyl groups found in cellulose, pectin, and absorbed water. The peak at 2925 cm^−1^ is due to the C-H stretching of aliphatic methylene groups within the polysaccharide backbones. A sharp and distinguishing peak observed at 1738 cm^−1^ is attributed to the C=O stretching of esterified carboxyl groups, a hallmark feature of pectin. The adjacent peak at 1620 cm^−1^ can be assigned to the C=O stretching of dissociated carboxylate groups (COO^−^) and may also include contributions from aromatic C=C vibrations in lignin. Finally, the complex “fingerprint” region between 1000 and 1200 cm^−1^ contains multiple overlapping peaks corresponding to C-O and C-C stretching, as well as C-O-C glycosidic bond vibrations within the cellulose and pectin structures.

#### 3.2.2. XRD Analysis: Crystallinity and Mineral Phases

**Figure 2:**
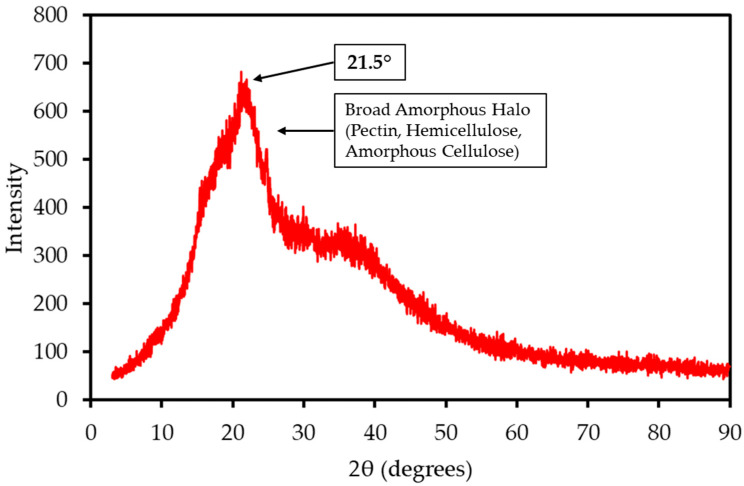
XRD pattern of LP sample. The pattern is dominated by a broad amorphous halo characteristic of non-crystalline pectin and hemicellulose, with a weak superimposed peak corresponding to the crystalline structure of cellulose I.

The XRD pattern of LP, shown in Figure 2, is dominated by a broad amorphous halo centered at a 2θ angle of approximately 21.5°. This pattern is characteristic of the largely non-crystalline structures of pectin, hemicellulose, and amorphous cellulose. A very weak, broad crystalline peak, corresponding to the (002) plane of cellulose I, is superimposed on this halo. The Crystallinity Index (CrI) was calculated to be approximately 34%. This low degree of structural order is common in pectin-rich biomass and suggests that the material can be more readily accessed and decomposed during thermal treatment compared to highly crystalline woody biomass. In contrast to the mango peel analyzed in the previous study, which exhibited a distinct crystalline peak for calcite [22], the LP sample shows no sharp mineral peaks. This suggests that its inorganic components are finely dispersed throughout the biomass matrix or exist in an amorphous state.

#### 3.2.3. X-Ray Fluorescence (XRF) of Ash

The composition of the ash produced from LP combustion was determined by XRF analysis, with the results presented in Table 2. The ash is composed of alkali and alkaline earth metals, which has critical implications for combustion applications. Potassium oxide (K_2_O, 34.8 wt%) and calcium oxide (CaO, 31.5 wt%) are the dominant components, together constituting over 66% of the ash mass. This high concentration of potassium is a major concern, as it is known to lower the ash fusion temperature, leading to severe slagging, fouling, and corrosion issues in boilers. This finding is consistent with the high potassium content that was also observed in mango peel ash [22]. This corrosion risk can be worsened by the chlorine content. The chlorine in the ash was found to be 5.24 wt%, which corresponds to a total chlorine concentration of approximately 0.28 wt% in the original dry biomass. This level is considered significant and can contribute to high-temperature corrosion and fouling through the formation of alkali chlorides, especially in the presence of the abundant potassium. Although the high calcium content can sometimes help to capture sulfur and increase the ash melting point, the overall high concentration of fluxing agents requires careful management. The ash also contains significant amounts of silica (SiO_2_, 10.8 wt%) and sulfur (SO_3_, 4.25 wt%). While the high potassium content poses slagging and fouling risks during combustion, it also offers opportunities for ash reuse as a potassium-rich fertilizer or soil amendment, potentially contributing to sustainable agriculture by recycling nutrients and improving soil fertility in potassium-deficient areas [10]. For instance, similar high-K ashes from biomass have been shown to increase crop biomass by over three-fold when used as alternatives to conventional fertilizers [31]. These results confirm that any industrial application of LP as a fuel would necessitate co-firing with a low-ash fuel or using combustion systems specifically designed to handle high-fouling biomass.

#### 3.2.4. SEM Analysis: Heterogeneous Surface Morphology

**Figure 3:**
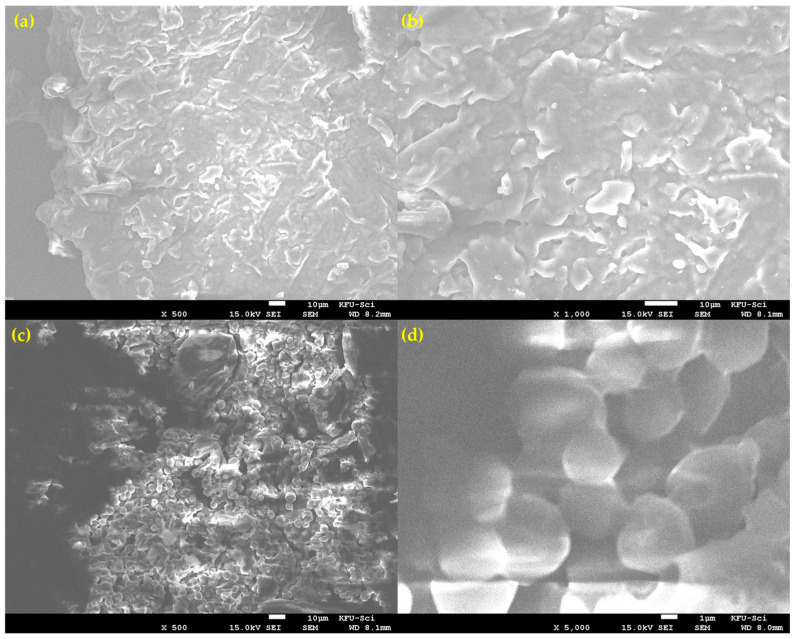
SEM micrographs showing the heterogeneous morphology of the prepared lemon peel powder. (**a**) Overview of a large, fibrous particle type. (**b**) Close-up of the flaky, layered surface of a fibrous particle. (**c**) Region showing the second dominant morphology of agglomerated, globular particles. (**d**) High-magnification view of the semi-fused and porous globular structure.

Scanning Electron Microscopy (SEM) revealed that the milled lemon peel (LP) powder is morphologically heterogeneous, consisting of at least two distinct particle types (Figure 3). The first morphology, shown in Figure 3a,b, consists of larger, fibrous particles with a flaky and layered surface texture. These particles appear to correspond to the more rigid structural components of the peel. The second, more dominant morphology, seen in Figure 3c,d, is characterized by agglomerates of smaller, semi-fused globular particles. At high magnification (Figure 3d), this structure is clearly porous. This observed heterogeneity is critical for understanding the combustion process. The presence of different particle shapes, sizes, and porosities means that heat and mass transfer characteristics will vary throughout the bulk sample. This physical complexity strongly supports the kinetic analysis finding that the overall reaction is governed by a three-dimensional diffusion-controlled mechanism (D3), as the reaction rate is averaged over these diverse structures.

### 3.3. Combustion Characteristics via TGA

**Figure 4:**
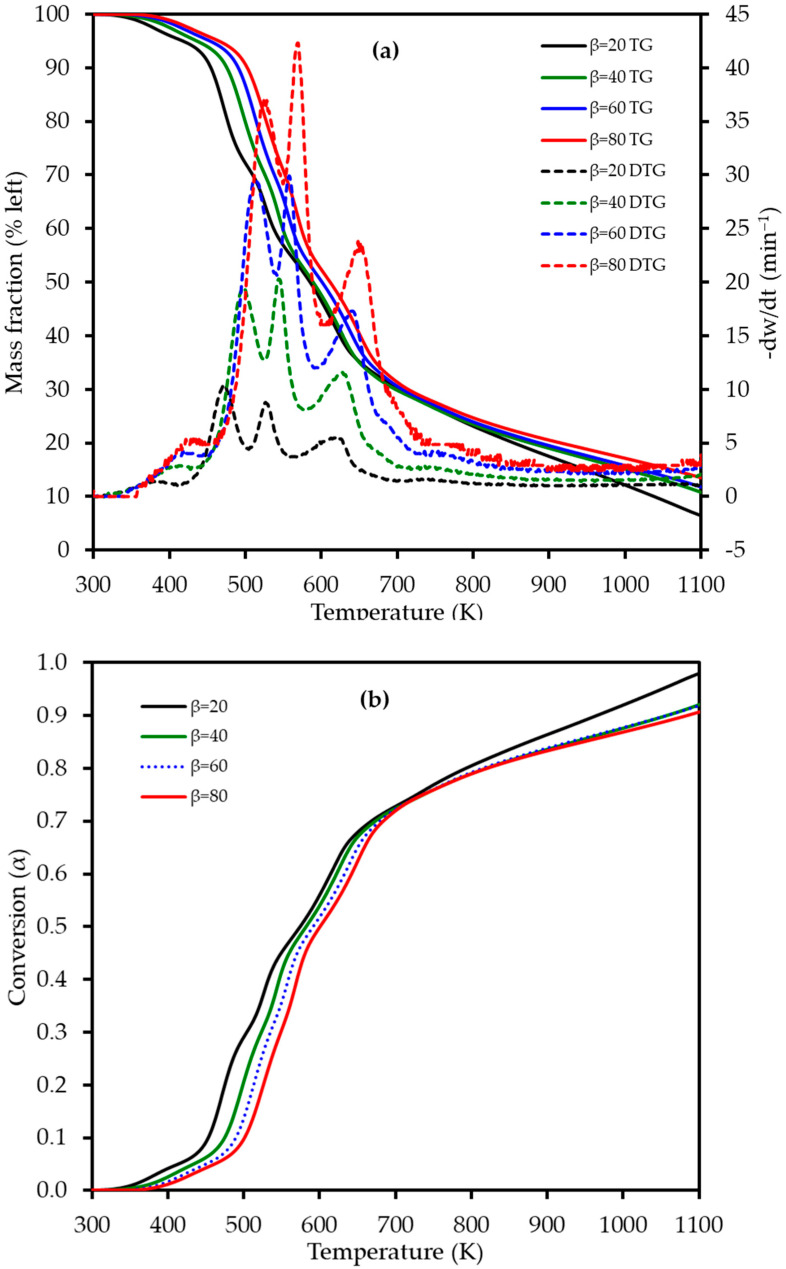
(**a**) Thermogravimetric (TG) (left axis) and derivative thermogravimetric (DTG) (right axis) curves and (**b**) conversion (α) profiles for lemon peel (LP) combustion at four different heating rates (β): 20, 40, 60, and 80 K min^−1^. The TG curves represent the average of triplicate measurements.

The thermogravimetric (TG) and derivative thermogravimetric (DTG) profiles for LP combustion at heating rates of 20, 40, 60, and 80 K min^−1^ are presented in Figure 4. As the heating rate was increased, a consistent shift in both the TG and DTG curves to higher temperatures was observed. This phenomenon is attributed to thermal lag; at higher heating rates, the system has less time to reach thermal equilibrium, leading to a delay in the onset of decomposition reactions. This multi-stage degradation is characteristic of lignocellulosic materials, though the specific temperatures under oxidative conditions differ from those reported in pyrolysis studies of LP and other citrus wastes [18,32].

The DTG curves clearly reveal four distinct, albeit overlapping, stages of mass loss during the combustion process, which are detailed in Table 3. This four-stage decomposition profile for lemon peel differs from the three-stage profile observed for mango peel [22], likely due to the high pectin content in LP. The first stage, occurring between 340 and 460 K, corresponds to dehydration, with a mass loss of approximately 4–5% as moisture is released from the sample. This was followed by the primary devolatilization in Stage 2 (410–550 K), which exhibited a significant mass loss of 25–27% and is attributed to the degradation of the most thermally labile components, primarily pectin and hemicellulose. The third stage (505–610 K) involved a further mass loss of 18–21% and is mainly associated with the decomposition of cellulose. The process concluded with the fourth stage (565–740 K), which represents the slow decomposition of the more thermally stable lignin fraction, followed by the oxidation of the resulting fixed carbon (char), with a mass loss ranging from 22 to 26%. The high heating rates explored in this study revealed intensified thermal behavior not captured by previous studies that focused on pyrolysis at lower rates. No prior studies appear to have reported on the multi-stage combustion behavior of lemon peel using TGA, highlighting the novelty of these findings for understanding its potential in industrial thermal processing.

**Figure 5:**
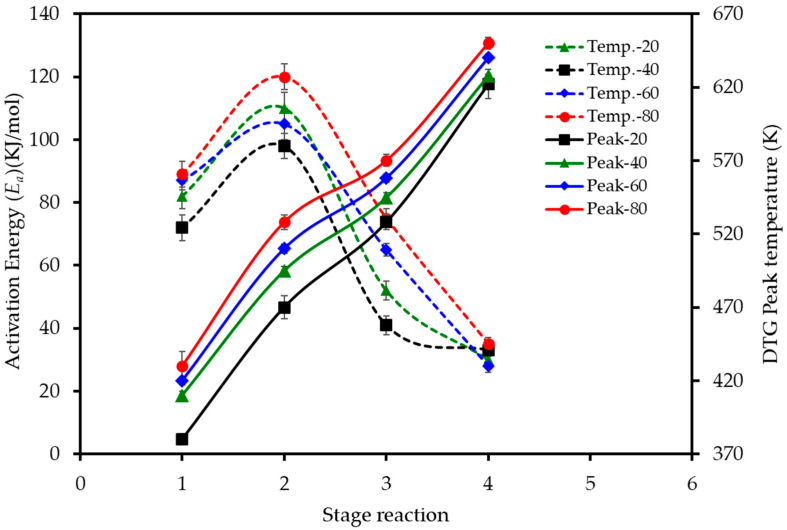
Effect of heating rate on the activation energy and peak temperatures for the main stages of LP combustion. Dashed lines (left axis) show the activation energy (*E_a_*) calculated using the D3 model for each stage. Solid lines (right axis) show the peak reaction temperature (*T_peak_*) for each stage, as determined from the DTG curves.

The effect of the heating rate on the decomposition stages is further visualized in Figure 5. The data reveals two key trends. Firstly, the activation energy (*E_a_*) required for each stage varies significantly, with the second stage of devolatilization (pectin/hemicellulose degradation) consistently demanding the highest energy input across all heating rates. Secondly, the peak temperature for each of the four combustion stages shifts to higher values as the heating rate is increased from 20 to 80 K min^−1^, confirming the presence of thermal lag in the system.

### 3.4. Kinetic Modelling of Lemon Peel Combustion

#### 3.4.1. Activation Energies from Model-Free Methods

**Figure 6:**
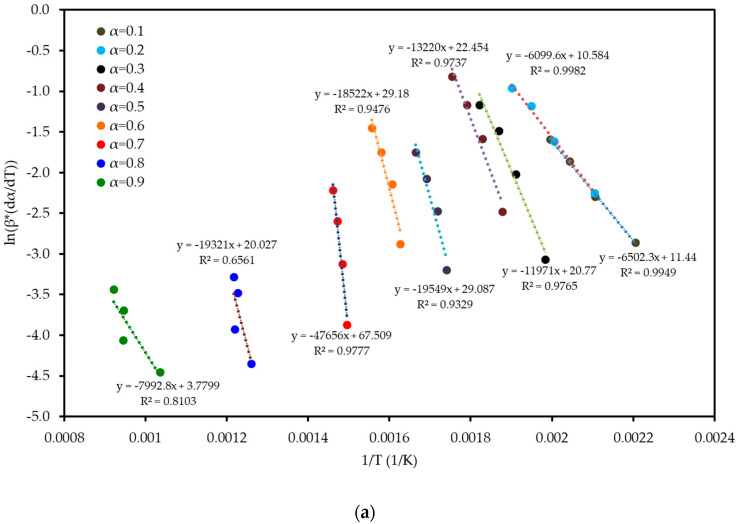

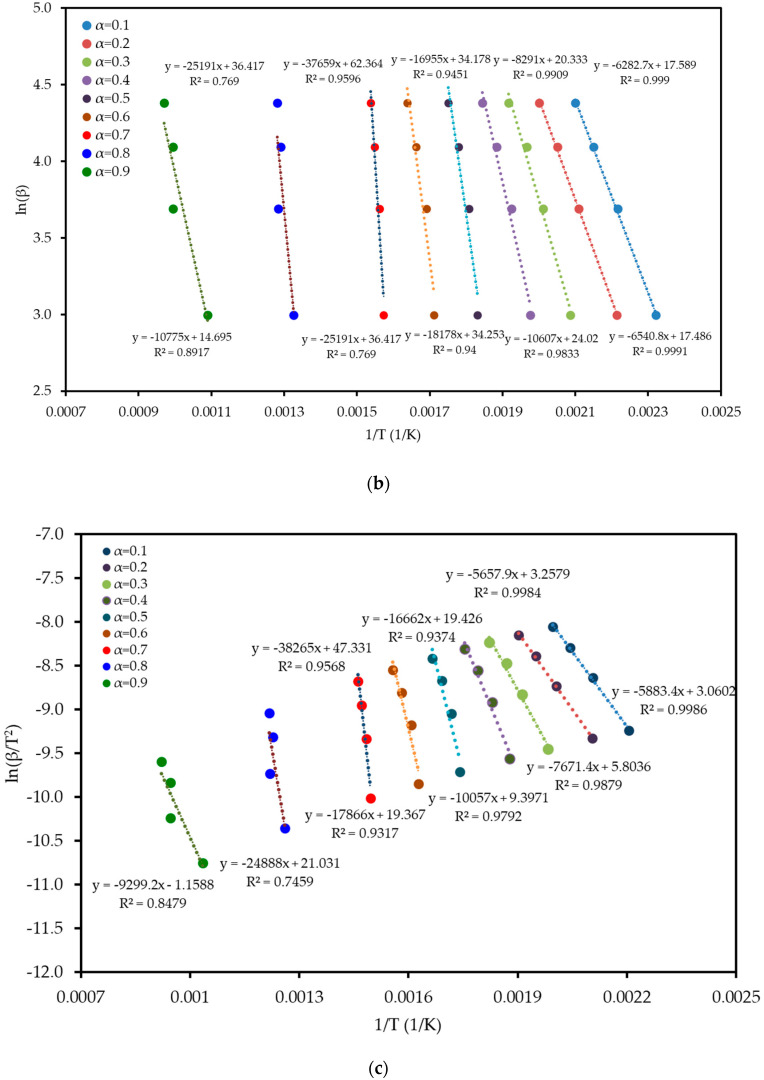

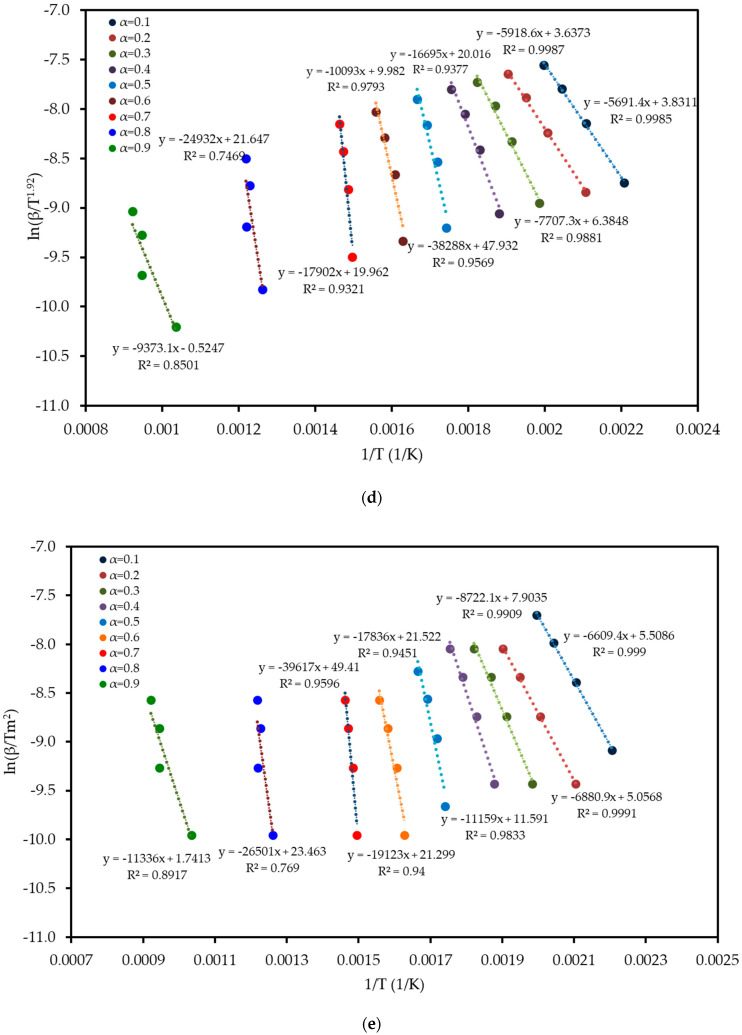
Regression plots for the linear isoconversional (model-free) methods used to determine the activation energy of LP combustion: (**a**) Friedman (FR), (**b**) Flynn–Wall–Ozawa (FWO), (**c**) Kissinger–Akahira–Sunose (KAS), (**d**) Starink (STK), and (**e**) Kissinger (K). The high linearity of the fits across multiple conversion (α) levels validates the application of these methods.

**Figure 7:**
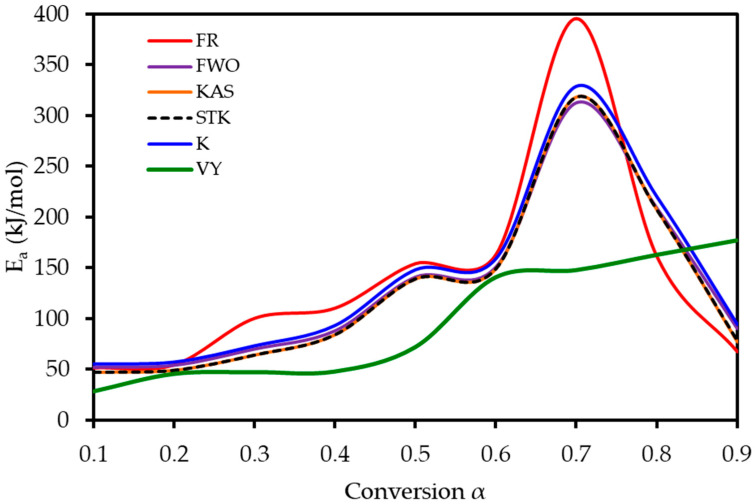
Activation energy (*E_a_*) as a function of conversion (α) for lemon peel combustion, as determined using six model-free methods.

To understand the complex energy requirements of the multi-step combustion process, the apparent activation energy (*E_a_*) was determined as a function of conversion (α) using six independent, model-free (isoconversional) methods. These methods, which are widely endorsed for kinetic analysis of complex solids, do not assume a predefined reaction mechanism and rely on data from multiple heating rates. The linear regression plots used to calculate *E_a_* for five of these methods are shown in Figure 6, and the final *E_a_* values are compiled in Table 4 and visualized in Figure 7. The results in Figure 7 clearly show that the activation energy is not constant but varies significantly throughout the reaction, confirming the complexity of LP combustion. The process begins with a relatively low *E_a_* of approximately 47 kJ mol^−1^ (at α = 0.1), corresponding to the initial breakdown of the most thermally labile components, such as pectin and hemicellulose. As the conversion progresses, the *E_a_* steadily increases, representing the decomposition of more stable structures like cellulose and the formation of a complex char matrix. The energy demand reaches a distinct peak of 304 kJ mol^−1^ at a conversion of approximately α = 0.7, which aligns with the temperature range for the degradation of lignin, the most recalcitrant of the lignocellulosic components. Beyond this peak, the *E_a_* declines, suggesting that the final burnout of the remaining char is a less energy-intensive process. The various model-free methods produced highly consistent results, lending confidence to the analysis. The integral methods (FWO, KAS, STK, K) yielded very similar and overlapping *E_a_* curves. The differential Friedman (FR) method followed the same trend but produced a higher peak *E_a_* value (396 kJ mol^−1^), a common characteristic as this method is more sensitive to fluctuations in the DTG signal. This distinction between the linear approximation methods (FWO, KAS, STK) and the more accurate non-linear methods like Vyazovkin is a key consideration in kinetic analysis and justifies their separate evaluation [33]. The high correlation coefficients (R^2^ > 0.93) across the main conversion range (α = 0.1–0.7) in Table 4 confirm the excellent quality of the linear fits. The observed decrease in R^2^ at very high conversions (α ≥ 0.8) is expected and attributed to the diminished reaction rate and lower signal-to-noise ratio during the final char burnout phase.

#### 3.4.2. Reaction Mechanism and Kinetic Parameters from CR Method

To identify the most probable solid-state reaction mechanism governing the combustion, the Coats–Redfern (CR) model-fitting method was employed. The kinetic data from each of the four decomposition stages (identified in Table 3) were fitted to sixteen different solid-state reaction models at each of the four heating rates. The resulting activation energies (*E_a_*), pre-exponential factors (*lnA*_0_), and correlation coefficients (R^2^) are summarized in Table 5. Across all four combustion stages and at all heating rates, the three-dimensional diffusion model (D3) consistently provided the best statistical fit to the experimental data, yielding the highest correlation coefficients (R^2^ > 0.99 for many stages). This result strongly indicates that the overall rate of LP combustion is governed by a diffusion-controlled mechanism. In this type of process, the reaction rate is limited not by the chemical kinetics but by the physical transport of reactant gases (i.e., oxygen) through the porous char layer to the reaction surface and the subsequent escape of product gases. Due to its superior statistical fit, the D3 model was selected as the most appropriate mechanism to describe the LP combustion process. The determination of a diffusion-controlled mechanism is physically consistent with the combustion of a porous solid fuel and aligns with findings for other citrus peels [34] as well as our previous work on mango peel [22]. This mechanism is a direct reflection of the physical transformations the LP particles undergo as they are converted into a porous char matrix. The observed morphological heterogeneity, with both fibrous and porous globular structures (Figure 3), reinforces this conclusion, as a diffusion-limited model naturally accounts for the averaged combustion behavior of these physically distinct particle types.

### 3.5. Thermodynamic Assessment of Combustion

To further evaluate the feasibility and energy dynamics of lemon peel combustion, thermodynamic parameters, including activation enthalpy (Δ*H*), Gibbs free energy (Δ*G*), and entropy (Δ*S*), were calculated from the kinetic parameters. The results, derived from the kinetic parameters obtained via the model-free methods, are compiled in Table 5. The calculated values for activation enthalpy (Δ*H*) are positive across all conversion levels, confirming that the formation of the activated complex from the reactants is an endothermic process. This means that energy is required from the system to initiate the reaction steps, even though the overall combustion of the biomass is an exothermic process. The difference between the activation energy (*E_a_*) and the activation enthalpy (Δ*H*) was found to be consistently small (in the range of 3.5–5.4 kJ mol^−1^), which implies that only a modest additional energy barrier must be overcome to convert the activated complex into reaction products, thus facilitating the overall reaction once sufficient temperature is reached.

The Gibbs free energy (Δ*G*) was also found to be positive for all methods across the entire conversion range (81.82–220.08 kJ mol^−1^), indicating that the combustion process is non-spontaneous. The high positive values, particularly at higher conversions, signify a substantial thermodynamic barrier that requires a continuous supply of external thermal energy to sustain the reaction, especially during the later char oxidation stages. The entropy (Δ*S*) values reveal a notable transition during the combustion process. At low to mid-levels of conversion, the Δ*S* values are predominantly negative, suggesting that the activated complex is more structured and ordered than the initial reactants. This corresponds to the initial devolatilization phase where complex biopolymers are breaking down into a more organized nascent char structure. In contrast, at high conversions (α ≥ 0.7), the Δ*S* values become positive, reflecting an increase in system disorder as the solid char matrix is broken down into a large volume of gaseous products. This behavior is characteristic of the complex thermal degradation of lignocellulosic fuels and underlines the interplay between kinetic and thermodynamic factors in LP combustion, which proceeds via an endothermic devolatilization phase followed by diffusion-controlled char oxidation.

### 3.6. Integrated Analysis and Implications for Biofuel Applications

This comprehensive analysis demonstrates that lemon peel (LP) is a high-potential but challenging solid biofuel. Its primary advantages are its widespread availability as a waste product and its excellent fuel properties, including a high volatile matter content (73.20 wt%), a complex and heterogeneous particle morphology conducive to thermal decomposition, and an exceptionally high energy content (HHV = 23.02 MJ kg^−1^), which surpasses that of many other agricultural residues [29,31]. Thermogravimetric analysis revealed a four-stage decomposition process, and kinetic modeling confirmed that the combustion is kinetically feasible, governed by a three-dimensional diffusion-controlled mechanism (D3) with an average activation energy of ~126 kJ mol^−1^.

However, the practical application of LP as a standalone fuel requires careful consideration of its ash composition. The ash analysis revealed an extremely high concentration of potassium oxide (34.8 wt% of the ash), which poses a severe risk of slagging, fouling, and corrosion in conventional combustion systems [29,30]. This remains the primary challenge for its use as a biofuel. From a circular economy viewpoint, the potassium-rich ash could be valorized as a bio-fertilizer, offsetting waste disposal costs and creating an additional value stream while mitigating environmental impacts through nutrient recycling. In contrast to this challenge, a key advantage identified in the ultimate analysis is the low nitrogen (1.26 wt%) and sulfur (0.20 wt%) content. This makes LP an environmentally attractive fuel, as it would be expected to produce significantly lower NO_x_ and SO_x_ emissions compared to coal or other nitrogen-rich biomass types. Therefore, while LP is a potent and relatively clean energy source from an emissions perspective, its direct combustion is not recommended without strategies to mitigate ash-related issues. Viable pathways for its utilization would include: (1) co-firing in small percentages with a primary fuel that has low ash and alkali content (e.g., natural gas or clean woody biomass) to dilute the problematic elements; or (2) utilizing specialized combustion technologies, such as fluidized bed reactors, which are better equipped to handle high-fouling fuels. These findings highlight that complete fuel characterization, weighing both advantages (high HHV, low N/S) and disadvantages (high K and Cl), is critical for evaluating any biofuel. Furthermore, these challenges with direct use highlight why alternative valorization routes, such as pyrolyzing the peel to produce an upgraded biochar fuel as explored by Selvarajoo et al. (2022) [18], remain an active area of research. Indeed, the future of citrus peel valorization lies in a holistic biorefinery approach, where direct combustion is one of several potential pathways alongside the production of biochemicals, bio-adsorbents, and other value-added products within a circular bioeconomy framework (Kumar et al., 2025) [5].

## 4. Conclusions

This study successfully conducted a comprehensive investigation into the combustion of lemon peel (LP) at high, industrially relevant heating rates (20–80 K min^−1^). The evaluation of LP’s viability as a solid biofuel reveals both significant potential and considerable drawbacks. From an energy perspective, it is a highly promising feedstock, possessing a high heating value of 23.02 MJ kg^−1^, complemented by low nitrogen (1.26 wt%) and sulfur (0.20 wt%) content, which suggests a lower environmental impact from NOₓ and SOₓ emissions. The thermal decomposition of LP proceeds via four distinct stages, and a rigorous kinetic analysis revealed that the activation energy is highly dependent on conversion, peaking at approximately 304 kJ mol^−1^. The Coats–Redfern method confirmed that a three-dimensional diffusion (D3) mechanism is the rate-limiting step. However, the fuel’s potential is challenged by its problematic ash chemistry, where a high potassium oxide content (34.8 wt% K_2_O) poses a significant slagging and fouling risk. This challenge also presents a circular economy opportunity, as the ash could be valorized as a bio-fertilizer. Therefore, while LP is a potent and relatively clean energy source, its practical implementation requires advanced ash management strategies. Future work should focus on pilot-scale trials to test ash mitigation techniques, such as co-firing or the use of additives, and explore alternative valorization pathways to fully unlock the potential of this abundant bioresource.

## Figures and Tables

**Table 1:** Characteristics of the LP sample studied in this work.

Analysis/Property	Parameter	Value
Proximate Analysis		
	Moisture Content	9.73 ± 0.02
	Volatile Matter	73.20 ± 0.11
	Ash	5.30 ± 0.03
	Fixed Carbon ^a^	11.70 ± 0.10
Ultimate Analysis (dry basis)		
	Carbon (C)	54.09 ± 0.16
	Hydrogen (H)	4.95 ± 0.03
	Nitrogen (N)	1.26 ± 0.03
	Sulfur (S)	0.20 ± 0.04
	Oxygen (O) ^b^	34.20 ± 0.18
Heating Value (MJ kg^−1^)		23.02 ± 0.05
Fiber Fraction (dry basis)		
	Hemicellulose	0.73 ± 0.05
	Cellulose	10.49 ± 0.35
	Lignin	0.78 ± 0.11

^a^ Fixed carbon is calculated by difference: 100 − (Moisture + Volatile Matter + Ash) %. ^b^ Oxygen content is determined by difference: 100 − (C + H + N + S + Ash) %.

**Table 2:** Ash composition of LP by XRF Analysis.

Component	Oxide (wt%)	Elemental (wt%)
K_2_O	34.75	K: 28.85
CaO	31.51	Ca: 22.52
SiO_2_	10.78	Si: 5.04
Al_2_O_3_	6.86	Al: 3.63
Cl	5.24	Cl: 5.24
SO_3_	4.25	S: 1.70
P_2_O_5_	2.77	P: 1.21
Fe_2_O_3_	1.5	Fe: 1.05
Others	<1.0	<1.0

**Table 3:** Characteristic temperatures and weight losses (%) for LP combustion.

Heating Rate (K min^−1^)	1st Reaction			2nd Reaction			3rd Reaction			4th Reaction		
	T Range, T Peak (K)	Weight Loss %	Process	T Range, T Peak (K)	Weight Loss %	Process	T Range, T Peak (K)	Weight Loss %	Process	T Range, T Peak (K)	Weight Loss %	Process
20	340–410, 380	5	dehydration	410–505, 470	25	Pectin/Hemicellulose degradation	505–565, 528	18	Cellulose degradation	565–700, 622	26	Lignin degradation
40	340–430, 410	5	dehydration	430–525, 495	25	Pectin/Hemicellulose degradation	525–580, 545	20	Cellulose degradation	580–700, 628	22	Lignin degradation
60	340–440, 420	4	dehydration	440–540, 510	27	Pectin/Hemicellulose degradation	540–590, 558	19	Cellulose degradation	590–720 640	24	Lignin degradation
80	340–460, 430	5	dehydration	460–550, 528	25	Pectin/Hemicellulose degradation	550–610, 570	21	Cellulose degradation	610–740, 650	24	Lignin degradation

**Table 4:** Kinetic parameter values obtained using six model-free methods for LP combustion at different conversion levels.

Conversion	FR		FWO		KAS		STK		K		VY		Average ^b^	
	E (kJ mol)	R^2^	E (kJ/mol)	R^2^	E (kJ/mol)	R^2^	E (kJ/mol)	R^2^	E (kJ/mol)	R^2^	E (kJ/mol)	R^2^	E (kJ/mol)	R^2^
0.1	51	0.9982	52	0.999	47	0.9984	47	0.9985	55	0.999	29	NA ^a^	50	0.9986
0.2	54	0.9949	54	0.9991	49	0.9986	49	0.9987	57	0.9991	46	NA	53	0.9981
0.3	100	0.9765	70	0.9909	64	0.9879	64	0.9881	73	0.9909	47	NA	74	0.9869
0.4	110	0.9737	88	0.9833	84	0.9792	84	0.9793	93	0.9833	48	NA	92	0.9798
0.5	154	0.9329	141	0.9451	139	0.9374	139	0.9377	148	0.9451	72	NA	144	0.9396
0.6	163	0.9476	151	0.94	149	0.9317	149	0.9321	159	0.94	141	NA	154	0.9383
0.7	396	0.9777	313	0.9596	318	0.9568	318	0.9569	329	0.9596	148	NA	335	0.9621
0.8	161	0.6561	209	0.769	207	0.7459	207	0.7469	220	0.769	163	NA	201	0.7374
0.9	67	0.8103	90	0.8917	77	0.8479	78	0.8501	94	0.8917	177	NA	81	0.8583
Average	140	0.9187	130	0.9420	126	0.9315	126	0.9320	136	0.9420	97	NA	132	0.9332

^a^ NA: R^2^ is not applicable as the Vyazovkin method is a non-linear isoconversional method that does not rely on linear regression. ^b^ The average *E_a_* is calculated from the five linear isoconversional methods (FR, FWO, KAS, STK, and K). The result from the advanced non-linear Vyazovkin (VY) method is presented in Figure 7 for a comparison but is excluded from this average due to its different mathematical basis.

**Table 5:** Pre-exponential factor and thermodynamic parameters of LP combustion.

**α**	**FR**					**FWO**				
	**R^2^**	***A*_0_, min** ^−1^	**Δ*H*** **(kJ mol^−1^)**	**Δ*G*** **(kJ mol^−1^)**	**Δ*S*** **(kJ mol^−1^ K^−1^)**	**R^2^**	***A*_0_, min^−1^**	**Δ*H*** **(kJ mol^−1^)**	**Δ*G*** **(kJ mol^−1^)**	**Δ*S*** **(kJ mol^−1^ K^−1^)**
0.1	0.9982	9.75 × 10^2^	47.51	131.03	−0.19886	0.999	1.71 × 10^9^	48.51	81.82	−0.07931
0.2	0.9949	5.16 × 10^3^	49.84	143.07	−0.18646	0.9991	6.42 × 10^9^	49.84	84.73	−0.06977
0.3	0.9765	9.93 × 10^7^	95.43	153.30	−0.10522	0.9909	2.09 × 10^11^	65.43	88.32	−0.04162
0.4	0.9737	8.28 × 10^8^	105.43	153.60	−0.08759	0.9833	1.29 × 10^13^	83.43	87.45	−0.00731
0.5	0.9329	9.36 × 10^11^	149.43	165.45	−0.02914	0.9451	3.62 × 10^17^	136.43	93.63	0.07781
0.6	0.9476	1.52 × 10^12^	158.43	172.24	−0.02511	0.94	5.92 × 10^17^	146.43	101.37	0.081914
0.7	0.9777	1.02 × 10^29^	390.60	198.42	0.295663	0.9596	7.28 × 10^29^	307.60	104.82	0.311969
0.8	0.6561	4.03 × 10^8^	155.60	217.32	−0.09496	0.769	9.27 × 10^18^	203.60	136.39	0.103394
0.9	0.8103	7.26 × 10^1^	61.60	207.25	−0.22408	0.8917	1.32 × 10^10^	84.60	127.47	−0.06596
**α**	**KAS**					**STK**				
	**R^2^**	***A*** **_0_** **, min^−1^**	**Δ*H*** **(kJ mol^−1^)**	**Δ*G*** **(kJ mol^−1^)**	**Δ*S*** **(kJ mol^−1^ K^−1^)**	**R^2^**	***A*** **_0_** **, min^−1^**	**Δ*H*** **(kJ mol^−1^)**	**Δ*G*** **(kJ mol^−1^)**	**Δ*S*** **(kJ mol^−1^ K^−1^)**
0.1	0.9984	1.75 × 10^2^	43.51	133.02	−0.21313	0.9985	9.72 × 10^0^	43.51	143.12	−0.23717
0.2	0.9986	6.46 × 10^2^	44.84	146.71	−0.20373	0.9987	3.31 × 10^1^	44.84	159.06	−0.22842
0.3	0.9879	3.21 × 10^4^	59.43	154.06	−0.17206	0.9881	9.68 × 10^2^	59.43	170.07	−0.20116
0.4	0.9792	2.99 × 10^6^	79.43	153.33	−0.13436	0.9793	5.25 × 10^4^	79.43	171.80	−0.16796
0.5	0.9374	1.94 × 10^11^	134.43	157.64	−0.04221	0.9377	1.25 × 10^9^	134.43	180.70	−0.08414
0.6	0.9317	3.20 × 10^11^	144.43	165.37	−0.03807	0.9321	1.81 × 10^9^	144.43	189.04	−0.08111
0.7	0.9568	1.50 × 10^24^	312.60	180.56	0.203126	0.9569	8.50 × 10^16^	312.60	270.74	0.064388
0.8	0.7459	5.84 × 10^12^	201.60	211.55	−0.01531	0.7469	1.74 × 10^10^	201.60	242.97	−0.06365
0.9	0.8479	8.47 × 10^3^	71.60	191.53	−0.18451	0.8501	5.17 × 10^1^	72.60	220.08	−0.2269
**α**	**K**									
	**R^2^**	***A*** **_0_** **, min^−1^**	**Δ*H*** **(kJ mol^−1^)**	**Δ*G*** **(kJ mol^−1^)**	**Δ*S*** **(kJ mol^−1^ K^−1^)**					
0.1	0.999	1.63 × 10^6^	51.51	109.11	−0.13714					
0.2	0.9991	1.08 × 10^6^	52.84	123.87	−0.14205					
0.3	0.9909	2.38 × 10^7^	68.43	132.84	−0.11711					
0.4	0.9833	1.21 × 10^9^	88.43	134.87	−0.08444					
0.5	0.9451	3.96 × 10^13^	143.43	142.34	0.001986					
0.6	0.94	3.40 × 10^13^	154.43	154.03	0.000728					
0.7	0.9596	1.14 × 10^26^	323.60	168.18	0.239099					
0.8	0.769	4.10 × 10^14^	214.60	201.58	0.02003					
0.9	0.8917	6.45 × 10^4^	88.60	197.56	−0.16763					

## Data Availability

All data generated or analysed during this study are included in this published article.

## Supplementary Materials

The following supporting information can be downloaded at: https://www.mdpi.com/article/10.3390/polym17212830/s1.
