# Supplementary material for: Assessing Lemon Peel Waste as a Solid Biofuel: A Study of Its Combustion Behaviour, Kinetics, and Thermodynamics
Source: Polymers (Basel). 2025 Oct 23;17(21):2830. doi: 10.3390/polym17212830 (PMC12610777; doi:10.3390/polym17212830)
Supplement: Supplementary file 1 [file polymers-17-02830-s001.zip › polymers-3900752-supplementary.pdf]

polymers

# Supplementary Materials for

## Assessing Lemon Peel Waste as a Solid Biofuel: A Study of its Combustion Behaviour, Kinetics, and Thermodynamics

**Mohamed Anwar Ismail[1], Ibrahim Dubdub[2,*], Suleiman Mousa[2], Mohammed Al-Yaari[2], Majdi Ameen Alfaiad[2], Abdullah Alshehab[3]**

[1] Mechanical Engineering Department, King Faisal University, P.O. Box 380, Al-Ahsa 31982, Saudi Arabia.
[2] Chemical Engineering Department, King Faisal University, P.O. Box 380, Al-Ahsa 31982, Saudi Arabia
[3]Physics Department, King Faisal University, P.O. Box 380, Al-Ahsa 31982, Saudi Arabia
*Correspondence: idubdub@kfu.edu.sa; Tel.: +966-13-5896989

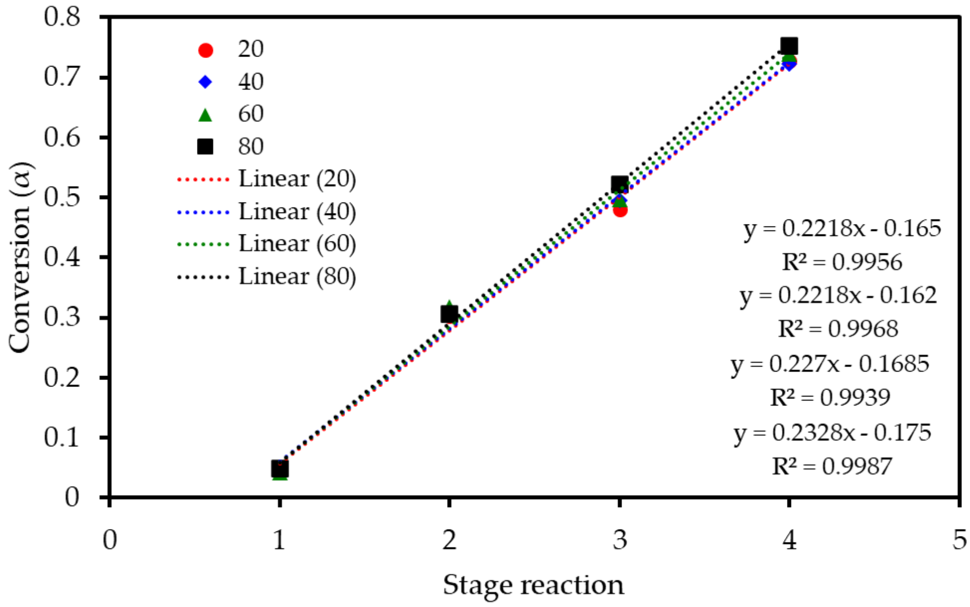


Figure S1. Cumulative Conversion at the End of Each Combustion Stage. The plot shows the total conversion ($\alpha$) reached after each of the four principal decomposition stages for all heating rates. The linear trendlines confirm the consistent, step-wise nature of the conversion process.

**Table S1.** Summary of model-free and model-fitting methods used for the kinetic analysis of MP combustion, including their corresponding equations and regression plots (Alhulaybi and Dubdub, 2024).

| model-free methods | | |
|---|---|---|
| **Method** | **Formula** | **Plot** |
| FR | $ln\left(\beta\frac{d\alpha}{dT}\right) = ln[A_o f(\alpha)] - \frac{E_a}{RT}$ (4) | $ln\left(\beta\frac{d\alpha}{dT}\right) vs. \frac{1}{T}$ |
| FWO | $ln(\beta) = ln\frac{A_o E_a}{Rg(\alpha)} - 5.331 - 1.052\frac{E_a}{RT}$ (5) | $ln(\beta)\ vs.\ \frac{1}{T}$ |
| KAS | $ln\left(\frac{\beta}{T^2}\right) = ln\frac{A_o R}{E_a\, g(\alpha)} - \frac{E_a}{RT}$ (6) | $ln\left(\frac{\beta}{T^2}\right) vs.\ \frac{1}{T}$ |
| STK | $ln\frac{\beta}{T^{1.92}} = ln\left(\frac{A_0 E_a}{Rg(\alpha)}\right) - 1.0008\frac{E_a}{RT}$ (7) | $ln\frac{\beta}{T^{1.92}}\ vs.\ \frac{1}{T}$ |
| K | $\ln\left(\frac{\beta}{T_m^2}\right) = ln\left(\frac{A_0 R}{E_a}\right) - \frac{E_a}{RT}$ (8) | $\ln\left(\frac{\beta}{T_m^2}\right) vs.\ \frac{1}{T}$ |
| VY | $\Phi(E_\alpha) = \sum_{i=1}^{n}\sum_{j\neq i}^{n}\frac{J[E_\alpha, T_i(t_\alpha)]}{J[E_\alpha, T_j(t_\alpha)]} = 0$ (9) | minimizing the function $\Phi(E_\alpha)$ |
| model-fitting methods | | |
| **Method** | **Formula** | **Plot** |
| CR | $ln\left[\frac{g(\alpha)}{T^2}\right] = ln\left[\frac{A_o R}{\beta E_a}\right] - \frac{E}{RT}$ (10) | $ln\left[\frac{g(\alpha)}{T^2}\right] vs. \frac{1}{T}$ |

**Table S2.** Fifteen of solid–state reaction mechanism (Alhulaybi and Dubdub, 2024 ).

| **Reaction mechanism** | **Code** | $f(\alpha)$ | $g(\alpha)$ |
|---|---|---|---|
| Reaction order models-1st order | F1 | 1-$\alpha$ | $-\ln(1-\alpha)$ |
| Reaction order models-2ndorder | F2 | $(1-\alpha)^2$ | $(1-\alpha)^{-1}-1$ |
| Reaction order models-3rd order | F3 | $(1-\alpha)^3$ | $[(1-\alpha)^{-2}-1]/2$ |
| Diffusion model-1 dimension | D1 | $1/2\alpha^{-1}$ | $\alpha^2$ |
| Diffusion model-2-dimension | D2 | $[-ln(1-\alpha)]^{-1}$ | $(1-\alpha)\ln(1-\alpha)+\alpha$ |
| Diffusion model-3-dimension | D3 | $3/2[1-(1-\alpha)^{1/3}]^{-1}$ | $[1-(1-\alpha)^{1/3}]^2$ |
| Nucleation models-2 dimension | A2 | $2(1-\alpha)[-\ln(1-\alpha)]^{1/2}$ | $[-\ln(1-\alpha)]^{1/2}$ |
| Nucleation models-3-dimension | A3 | $3(1-\alpha)[-\ln(1-\alpha)]^{1/3}$ | $[-\ln(1-\alpha)]^{1/3}$ |
| Nucleation models-4-dimension | A4 | $4(1-\alpha)[-\ln(1-\alpha)]^{1/4}$ | $[-\ln(1-\alpha)]^{1/4}$ |
| Geometrical contraction models-1-dimension | R1 | 1 | $\alpha$ |
| Geometrical contraction models - sphere | R2 | $2(1-\alpha)^{1/2}$ | 1-$(1-\alpha)^{1/2}$ |
| Geometrical contraction models - cylinder | R3 | $3(1-\alpha)^{1/3}$ | 1-$(1-\alpha)^{1/3}$ |
| Nucleation models-2-Power law | P2 | $2\alpha^{1/2}$ | $\alpha^{1/2}$ |

| | | | |
|---|---|---|---|
| Nucleation models-3-Power law | P3 | $3\alpha^{2/3}$ | $\alpha^{1/3}$ |
| Nucleation models-4-Power law | P4 | $4\alpha^{3/4}$ | $\alpha^{1/4}$ |

**Table S3.** Kinetic parameters obtained by the CR method for LP combustion at four heating rates.

| Reaction mechanism 1 step reaction | Code | 20 | | | 40 | | | 60 | | |
|---|---|---|---|---|---|---|---|---|---|---|
| | | $E_a$ (kJ/mol) | Ln($A_0$) | $R^2$ | $Ea$ (kJ/mol) | Ln($A_0$) | $R^2$ | $Ea$ (kJ/mol) | Ln($A_0$) | $R^2$ |
| Reaction order models-First order | F1 | 38 | 14.74 | 0.9973 | 33 | 17.77 | 0.9984 | 40 | 16.61 | 0.9984 |
| Reaction order models-Second order | F2 | 39 | 14.65 | 0.9974 | 33 | 17.63 | 0.9985 | 41 | 16.51 | 0.9985 |
| Reaction order models-Third order | F3 | 39 | 14.53 | 0.9975 | 34 | 17.5 | 0.9986 | 41 | 16.39 | 0.9986 |
| Diffusion models-One dimension | D1 | 82 | 19.11 | 0.9976 | 71 | 14.82 | 0.9987 | 86 | 19 | 0.9986 |
| Diffusion models-Two dimension | D2 | 82 | 18.5 | 0.9976 | 72 | 14.24 | 0.9987 | 86 | 18.39 | 0.9987 |
| **Diffusion models-Three dimension** | **D3** | 82 | 17.07 | 0.9977 | 72 | 12.83 | 0.9987 | 87 | 16.98 | 0.9987 |
| Diffusion models-Four dimension | D4 | 82 | 17.02 | 0.9977 | 72 | 12.77 | 0.9987 | 87 | 16.93 | 0.9987 |
| Nucleation models-Two dimension | A2 | 16 | 19.11 | 0.9959 | 13 | 20.92 | 0.9973 | 17 | 20.73 | 0.9976 |
| Nucleation models-Three-dimension | A3 | 9 | 20.28 | 0.9935 | 7 | 21.66 | 0.9949 | 9 | 21.75 | 0.996 |
| Nucleation models Fourth dimension | A4 | 5 | 20.56 | 0.9884 | 3 | 21.5 | 0.9879 | 5 | 21.99 | 0.9925 |
| Geometrical contraction models-One dimension phase boundary | R1 | 38 | 14.86 | 0.9971 | 32 | 17.89 | 0.9983 | 40 | 16.73 | 0.9983 |
| Geometrical contraction models -Contracting sphere | R2 | 38 | 15.49 | 0.9972 | 33 | 18.54 | 0.9984 | 40 | 17.37 | 0.9984 |
| Geometrical contraction models- Contracting cylinder | R3 | 38 | 15.88 | 0.9972 | 33 | 18.92 | 0.9984 | 40 | 17.75 | 0.9984 |
| Nucleation models-Power law | P2 | 16 | 19.17 | 0.9957 | 13 | 21 | 0.9972 | 17 | 20.79 | 0.9974 |
| Nucleation models-Power law | P3 | 9 | 20.32 | 0.9931 | 6 | 21.56 | 0.9945 | 9 | 21.79 | 0.9957 |
| Nucleation models-Power law | P4 | 5 | 20.59 | 0.9877 | 3 | 21.53 | 0.9866 | 5 | 22.02 | 0.992 |

| Reaction mechanism1 step reaction | Code | 80 | | |
|---|---|---|---|---|
| | | $E_a$ (kJ/mol) | Ln($A_0$) | $R^2$ |
| Reaction order models-First order | F1 | 41 | 16.99 | 0.9979 |
| Reaction order models-Second order | F2 | 41 | 16.86 | 0.998 |
| Reaction order models-Third order | F3 | 42 | 16.76 | 0.9981 |
| Diffusion models-One dimension | D1 | 88 | 19.23 | 0.9982 |

| | | | | |
|---|---|---|---|---|
| Diffusion models-Two dimension | D2 | 88 | 18.62 | 0.9982 |
| **Diffusion models-Three dimension** | **D3** | 89 | 17.21 | 0.9982 |
| Diffusion models-Four dimension | D4 | 89 | 17.15 | 0.9982 |
| Nucleation models-Two dimension | A2 | 17 | 21.07 | 0.9968 |
| Nucleation models-Three-dimension | A3 | 9 | 22.09 | 0.9948 |
| Nucleation models Fourth dimension | A4 | 5 | 22.33 | 0.9903 |
| Geometrical contraction models-One dimension phase boundary | R1 | 41 | 17.11 | 0.9978 |
| Geometrical contraction models -Contracting sphere | R2 | 41 | 17.74 | 0.9979 |
| Geometrical contraction models- Contracting cylinder | R3 | 41 | 18.13 | 0.9979 |
| Nucleation models-Power law | P2 | 17 | 21.14 | 0.9967 |
| Nucleation models-Power law | P3 | 9 | 22.14 | 0.9945 |
| Nucleation models-Power law | P4 | 6 | 22.55 | 0.9897 |

| **Reaction mechanism 2 step reaction** | **Code** | **20** | | | **40** | | | **60** | | |
|---|---|---|---|---|---|---|---|---|---|---|
| | | ***$E_a$* (kJ/mol)** | **Ln($A_0$)** | **$R^2$** | ***Ea* (kJ/mol)** | **Ln($A_0$)** | **$R^2$** | ***Ea* (kJ/mol)** | **Ln($A_0$)** | **$R^2$** |
| Reaction order models-First order | F1 | 52 | 12.3 | 0.9963 | 47 | 14.9 | 0.9974 | 50 | 15.01 | 0.9963 |
| Reaction order models-Second order | F2 | 56 | 12.37 | 0.9955 | 54 | 13.33 | 0.998 | 58 | 13.33 | 0.9973 |
| Reaction order models-Third order | F3 | 61 | 13.62 | 0.9947 | 61 | 13.56 | 0.9985 | 65 | 14.65 | 0.9981 |
| Diffusion models-One dimension | D1 | 105 | 23.42 | 0.9974 | 90 | 19.1 | 0.9972 | 95 | 20.1 | 0.9959 |
| Diffusion models-Two dimension | D2 | 107 | 23.45 | 0.9972 | 94 | 19.47 | 0.9974 | 100 | 20.54 | 0.9962 |
| **Diffusion models-Three dimension** | **D3** | 110 | 22.69 | 0.997 | 98 | 19.08 | 0.9976 | 105 | 20.24 | 0.9966 |
| Diffusion models-Four dimension | D4 | 108 | 22.19 | 0.9971 | 96 | 18.34 | 0.9975 | 101 | 19.43 | 0.9964 |
| Nucleation models-Two dimension | A2 | 22 | 18.29 | 0.9951 | 19 | 19.92 | 0.996 | 21 | 20.28 | 0.9945 |
| Nucleation models-Three-dimension | A3 | 12 | 19.96 | 0.9931 | 10 | 21.26 | 0.9935 | 11 | 21.68 | 0.9911 |
| Nucleation models Fourth dimension | A4 | 7 | 20.56 | 0.9895 | 6 | 21.73 | 0.9874 | 6 | 22.1 | 0.9836 |
| Geometrical contraction models-One dimension phase boundary | R1 | 49 | 13.31 | 0.9971 | 41 | 16.34 | 0.9965 | 43 | 16.56 | 0.995 |
| Geometrical contraction models -Contracting sphere | R2 | 51 | 13.52 | 0.9967 | 44 | 16.33 | 0.997 | 47 | 16.51 | 0.9957 |
| Geometrical contraction models- Contracting cylinder | R3 | 76 | 14.14 | 0.9966 | 45 | 16.49 | 0.9971 | 48 | 16.65 | 0.9959 |

| | | | | | | | | | | |
|---|---|---|---|---|---|---|---|---|---|---|
| Nucleation models-Power law | P2 | 21 | 18.78 | 0.996 | | | | 17 | 20.92 | 0.992 |
| Nucleation models-Power law | P3 | 11 | 20.23 | 0.9942 | | | | 9 | 22.05 | 0.9857 |
| Nucleation models-Power law | P4 | 6 | 20.68 | 0.9906 | | | | 4 | 22.12 | 0.9683 |

| **Reaction mechanism 2 step reaction** | **Code** | **80** | | |
|---|---|---|---|---|
| | | ***$E_a$* (kJ/mol)** | **Ln($A_0$)** | **$R^2$** |
| Reaction order models-First order | F1 | 58 | 14.04 | 0.9988 |
| Reaction order models-Second order | F2 | 64 | 14.14 | 0.9993 |
| Reaction order models-Third order | F3 | 72 | 15.97 | 0.9996 |
| Diffusion models-One dimension | D1 | 112 | 23.75 | 0.9985 |
| Diffusion models-Two dimension | D2 | 116 | 24.07 | 0.9987 |
| **Diffusion models-Three dimension** | **D3** | 120 | 23.62 | 0.9988 |
| Diffusion models-Four dimension | D4 | 117 | 22.92 | 0.9987 |
| Nucleation models-Two dimension | A2 | 25 | 20.05 | 0.9983 |
| Nucleation models-Three-dimension | A3 | 14 | 21.75 | 0.9974 |
| Nucleation models Fourth dimension | A4 | 8 | 22.34 | 0.9957 |
| Geometrical contraction models-One dimension phase boundary | R1 | 52 | 15.45 | 0.9981 |
| Geometrical contraction models -Contracting sphere | R2 | 55 | 15.45 | 0.9985 |
| Geometrical contraction models- Contracting cylinder | R3 | 58 | 15.66 | 0.9986 |
| Nucleation models-Power law | P2 | 22 | 20.68 | 0.9972 |
| Nucleation models-Power law | P3 | 12 | 22.1 | 0.9955 |
| Nucleation models-Power law | P4 | 6 | 22.43 | 0.9917 |

| **Reaction mechanism 3 step reaction** | **Code** | **20** | | | **40** | | | **60** | | |
|---|---|---|---|---|---|---|---|---|---|---|
| | | ***$E_a$* (kJ/mol)** | **Ln($A_0$)** | **$R^2$** | ***$E_a$* (kJ/mol)** | **Ln($A_0$)** | **$R^2$** | ***$E_a$* (kJ/mol)** | **Ln($A_0$)** | **$R^2$** |
| Reaction order models-First order | F1 | 24 | 18.76 | 0.9958 | 18 | 20.6 | 0.9876 | 31 | 18.95 | 0.9946 |
| Reaction order models-Second order | F2 | 33 | 16.82 | 0.9971 | 27 | 18.85 | 0.9912 | 43 | 16.55 | 0.9964 |
| Reaction order models-Third order | F3 | 43 | 14.51 | 0.9979 | 37 | 16.68 | 0.9931 | 56 | 13.7 | 0.9975 |
| Diffusion models-One dimension | D1 | 42 | 16.43 | 0.9958 | 32 | 19.39 | 0.9897 | 52 | 16.05 | 0.9941 |

| | | | | | | | | | | |
|---|---|---|---|---|---|---|---|---|---|---|
| Diffusion models-Two dimension | D2 | 47 | 16.05 | 0.9963 | 36 | 19.1 | 0.9906 | 58 | 15.45 | 0.9948 |
| **Diffusion models-Three dimension** | **D3** | 52 | 16.34 | 0.9967 | 41 | 19.5 | 0.9915 | 65 | 15.49 | 0.9954 |
| Diffusion models-Four dimension | D4 | 48 | 17.14 | 0.9964 | 38 | 20.25 | 0.9909 | 60 | 16.46 | 0.995 |
| Nucleation models-Two dimension | A2 | 8 | 21.04 | 0.9891 | 5 | 22.02 | 0.9504 | 11 | 21.92 | 0.9887 |
| Nucleation models-Three-dimension | A3 | 2 | 20.78 | 0.9395 | NA | NA | NA | 4 | 22.25 | 0.9653 |
| Nucleation models Fourth dimension | A4 | NA | NA | NA | NA | NA | NA | 1 | 9.52 | 0.6351 |
| Geometrical contraction models-One dimension phase boundary | R1 | 17 | 20.34 | 0.9932 | 11 | 21.92 | 0.9793 | 22 | 20.91 | 0.9911 |
| Geometrical contraction models -Contracting sphere | R2 | 20 | 20.28 | 0.9947 | 15 | 22.06 | 0.9844 | 26 | 20.67 | 0.9931 |
| Geometrical contraction models- Contracting cylinder | R3 | 21 | 20.4 | 0.9951 | 16 | 22.22 | 0.9857 | 28 | 20.76 | 0.9937 |
| Nucleation models-Power law | P2 | 4 | 21.31 | 0.9684 | 1 | 21.32 | 0.5926 | 6 | 22.47 | 0.972 |
| Nucleation models-Power law | P3 | NA | NA | NA | NA | NA | NA | NA | NA | NA |
| Nucleation models-Power law | P4 | NA | NA | NA | NA | NA | NA | 1 | 21.63 | 0.6547 |

| **Reaction mechanism 3 step reaction** | **Code** | **80** | | |
|---|---|---|---|---|
| | | $E_a$ **(kJ/mol)** | **Ln($A_0$)** | $R^2$ |
| Reaction order models-First order | F1 | 37 | 18.39 | 0.997 |
| Reaction order models-Second order | F2 | 48 | 15.97 | 0.9968 |
| Reaction order models-Third order | F3 | 63 | 13.71 | 0.9978 |
| Diffusion models-One dimension | D1 | 61 | 14.87 | 0.9964 |
| Diffusion models-Two dimension | D2 | 68 | 14.13 | 0.9969 |
| **Diffusion models-Three dimension** | **D3** | 75 | 14 | 0.9974 |
| Diffusion models-Four dimension | D4 | 70 | 15.08 | 0.9971 |
| Nucleation models-Two dimension | A2 | 14 | 21.96 | 0.9945 |
| Nucleation models-Three-dimension | A3 | 6 | 22.63 | 0.9867 |
| Nucleation models Fourth dimension | A4 | 2 | 22.29 | 0.94 |
| Geometrical contraction models-One dimension phase boundary | R1 | 26 | 20.57 | 0.9949 |
| Geometrical contraction models -Contracting sphere | R2 | 31 | 20.23 | 0.9962 |
| Geometrical contraction models- Contracting cylinder | R3 | 33 | 20.27 | 0.9965 |
| Nucleation models-Power law | P2 | 8 | 22.67 | 0.987 |

| | | | | |
|---|---|---|---|---|
| Nucleation models-Power law | P3 | 2 | 22.92 | 0.9295 |
| Nucleation models-Power law | P4 | NA | NA | NA |

| **Reaction mechanism 4 step reaction** | **Code** | **20** | | | **40** | | | **60** | | |
|---|---|---|---|---|---|---|---|---|---|---|
| | | ***$E_a$* (kJ/mol)** | **Ln($A_0$)** | **$R^2$** | ***$E_a$* (kJ/mol)** | **Ln($A_0$)** | **$R^2$** | ***$E_a$* (kJ/mol)** | **Ln($A_0$)** | **$R^2$** |
| Reaction order models-First order | F1 | 14 | 20.65 | 0.999 | 15 | 21.29 | 0.9982 | 12 | 22.02 | 0.9911 |
| Reaction order models-Second order | F2 | 27 | 18.26 | 0.9992 | 28 | 18.9 | 0.9974 | 27 | 19.61 | 0.9961 |
| Reaction order models-Third order | F3 | 43 | 14.98 | 0.9989 | 44 | 15.68 | 0.9969 | 45 | 16.1 | 0.9974 |
| Diffusion models-One dimension | D1 | 19 | 20.93 | 0.9982 | 21 | 21.44 | 0.9997 | 15 | 22.63 | 0.9905 |
| Diffusion models-Two dimension | D2 | 24 | 20.56 | 0.9989 | 26 | 21.05 | 0.9995 | 20 | 22.32 | 0.9931 |
| **Diffusion models-Three dimension** | **D3** | 30 | 20.69 | 0.9993 | 33 | 21.2 | 0.9992 | 28 | 22.52 | 0.9949 |
| Diffusion models-Four dimension | D4 | 26 | 21.61 | 0.9991 | 28 | 22.1 | 0.9994 | 23 | 23.42 | 0.9938 |
| Nucleation models-Two dimension | A2 | 2 | 21.02 | 0.9831 | 2 | 21.65 | 0.9839 | 1 | 21.69 | 0.5609 |
| Nucleation models-Three-dimension | A3 | NA | NA | NA | NA | NA | NA | NA | NA | NA |
| Nucleation models Fourth dimension | A4 | NA | NA | NA | NA | NA | NA | NA | NA | NA |
| Geometrical contraction models-One dimension phase boundary | R1 | 4 | 21.7 | 0.9899 | 5 | 22.48 | 0.9991 | 2 | 22.58 | 0.8854 |
| Geometrical contraction models -Contracting sphere | R2 | 9 | 22.15 | 0.9977 | 10 | 22.81 | 0.9986 | 7 | 23.46 | 0.9803 |
| Geometrical contraction models- Contracting cylinder | R3 | 10 | 22.27 | 0.9983 | 11 | 22.92 | 0.9985 | 8 | 23.59 | 0.9857 |
| Nucleation models-Power law | P2 | NA | NA | NA | NA | NA | NA | NA | NA | NA |
| Nucleation models-Power law | P3 | NA | NA | NA | NA | NA | NA | NA | NA | NA |
| Nucleation models-Power law | P4 | NA | NA | NA | NA | NA | NA | NA | NA | NA |

| **Reaction mechanism 4 step reaction** | **Code** | **80** | | |
|---|---|---|---|---|
| | | ***$E_a$* (kJ/mol)** | **Ln($A_0$)** | **$R^2$** |
| Reaction order models-First order | F1 | 16 | 22.03 | 0.9981 |
| Reaction order models-Second order | F2 | 29 | 19.61 | 0.9979 |
| Reaction order models-Third order | F3 | 46 | 16.4 | 0.9977 |
| Diffusion models-One dimension | D1 | 22 | 22.11 | 0.9994 |
| Diffusion models-Two dimension | D2 | 28 | 21.74 | 0.9992 |

| | | | | |
|---|---|---|---|---|
| **Diffusion models-Three dimension** | **D3** | 35 | 21.87 | 0.999 |
| Diffusion models-Four dimension | D4 | 30 | 22.78 | 0.9991 |
| Nucleation models-Two dimension | A2 | 3 | 22.77 | 0.9836 |
| Nucleation models-Three-dimension | A3 | NA | NA | NA |
| Nucleation models Fourth dimension | A4 | NA | NA | NA |
| Geometrical contraction models-One dimension phase boundary | R1 | 6 | 23.35 | 0.9979 |
| Geometrical contraction models -Contracting sphere | R2 | 10 | 23.49 | 0.9981 |
| Geometrical contraction models- Contracting cylinder | R3 | 12 | 23.69 | 0.9981 |
| Nucleation models-Power law | P2 | NA | NA | NA |
| Nucleation models-Power law | P3 | NA | NA | NA |
| Nucleation models-Power law | P4 | NA | NA | NA |

*NA: Not available.
